# Correction: Noguchi et al. Establishment and Characterization of NCC-PMP1-C1: A Novel Patient-Derived Cell Line of Metastatic Pseudomyxoma Peritonei. *J. Pers. Med.* 2022, *12*, 258

**DOI:** 10.3390/jpm13091383

**Published:** 2023-09-15

**Authors:** Rei Noguchi, Yuki Yoshimatsu, Yooksil Sin, Takuya Ono, Ryuto Tsuchiya, Hiroshi Yoshida, Tohru Kiyono, Yutaka Yonemura, Tadashi Kondo

**Affiliations:** 1 Division of Rare Cancer Research, National Cancer Center Research Institute, Tokyo 104-0045, Japan; renoguch@ncc.go.jp (R.N.); yyoshima@ncc.go.jp (Y.Y.); ishin@ncc.go.jp (Y.S.); takuono@ncc.go.jp (T.O.); rytsuchi@ncc.go.jp (R.T.); 2 Department of Orthopedic Surgery, Graduate School of Medicine, Chiba University, Chiba 263-8522, Japan; 3 Department of Diagnostic Pathology, National Cancer Center Hospital, Tokyo 104-0045, Japan; hiroyosh@ncc.go.jp; 4 Exploratory Oncology Research and Clinical Trial Center, National Cancer Center, Kashiwa 277-8577, Japan; tkiyono@east.ncc.go.jp; 5 NPO to Support Peritoneal Surface Malignancy Treatment, Japanese/Asian School of Peritoneal Surface Oncology, Kyoto 600-8189, Japan; y.yonemura@coda.ocn.ne.jp; 6 Peritoneal Surface Malignancy Center, Department of Regional Cancer Therapy, Kishiwada Tokushukai Hospital, Kishiwada 596-8522, Japan; 7 Peritoneal Surface Malignancy Center, Department of Regional Cancer Therapy, Kusatsu General Hospital, Shiga 525-8585, Japan

## Text Correction

There was an error in the original publication [1], SNP array data on copy number variations were conducted using the inappropriate cell line. In the Abstract, “amplifications and” was added to the sentence. In Section 2.5, Single Nucleotide Polymorphism Array, “(the parameter: alfa = 0.0001)” was added. In Section 3.2, Characteristics of NCC-PMP1-C1 Cells, “Figure 2A,B” was replaced with “Figure 2”; “partial amplifications of chromosomes 1, 2, 7, 8, 9, 11, 12, 15, 16, and 17” was added; “deletions of chromosomal arms 1p, 3p, 6p, 18q, and 19p” was replaced with “deletions of chromosomes 8, 10, 13, 14, 15, 17, and 20”; “Figure 2B–D” was removed; “and the amplifications in deletions, a cancer-related gene STK11 was were not identified in 19p (Figure 2C)” was removed; and “multiple amplifications and deletions, CNVs of cancer-related genes were not identified” was added. In paragraph 2 of Section 4, “Finally, the genetic profiles of PMP using SNP array analysis revealed loss of STK11, a well-known tumor suppressor gene.” was replaced with “Finally, the genetic profiles of PMP using SNP array analysis revealed multiple CNVs.”

Corrections have been made to Abstract, Section 2.5, Section 3.2, and Paragraph 2 in Section 4:

**Abstract:** Pseudomyxoma peritonei (PMP) is the intraperitoneal accumulation of mucus due to a mucinous tumor. PMP predominantly occurs in low-grade carcinomas. The incidence rate of PMP is one to two cases per million people per year. The standard therapy of PMP comprises complete cytoreductive surgery and hyperthermic intraperitoneal chemotherapy. PMP recurs in about 50% of patients, and 30–40% are unable to receive the standard treatment because of its invasiveness. Therefore, novel therapies are of the utmost necessity. For basic and pre-clinical research, patient-derived cell lines are essential resources. However, only two PMP cell lines have been reported. Thus, we established a novel PMP cell line from resected metastatic PMP tissue. The cell line, named NCC-PMP1-C1, was maintained for more than 5 months and was passaged 25 times. NCC-PMP1-C1 cells demonstrated multiple amplifications and deletions, slow growth, tumorigenic ability, and dissemination of tumor cells in nude mice. We also used NCC-PMP1-C1 cells to screen drugs, which demonstrated a significant response to daunorubicin HCl, homoharringtonine, mitomycin C, and ponatinib. The NCC-PMP1-C1 cell line is the first PMP cell line derived from metastasized tissue and is a potential resource for basic and pre-clinical research on metastasized PMP.

### 2.5. Single Nucleotide Polymorphism Array

Single nucleotide polymorphism (SNP) array genotyping was examined with Infinium OmniExpressExome-8 v. 1.4 BeadChip (Illumina, San Diego, CA, USA). Extraction of genomic DNA was conducted from the cultured cells derived from the tumor tissues, and the DNA was amplified. Amplified DNA was hybridized on array slides using an iScan system (Illumina, San Diego, CA, USA). The calculation of Log R ratios and B allele frequencies was performed using Genome Studio 2011.1, cnvPartition v3.2.0 (Illumina, San Diego, CA, USA), and KaryoStudio Data Analysis Software v. 1.0 (Illumina, San Diego, CA, USA). Annotation mapping was conducted using the human reference genome version hg19 (GRCh37). Analyses of the SNP array data were conducted using R software version 4.0.3 (R Foundation for Statistical Computing, http://www.R-project.org, accessed on 20 August 2023) and the R ‘DNAcopy’ package version 1.64.0 (Bioconductor, https://bioconductor.org/, accessed on 20 August 2023) [25] (the parameter: alfa = 0.0001). The whole-genome log 10 ratio (tumor/reference) value was smoothed, excluding chromosomes X and Y, and abnormal copy number regions were detected using the circular binary segmentation algorithm [26,27]. In the tumor cells, the definitions of amplifications and deletions were decided by regions with copy numbers >3 and <1, respectively. Genes that showed copy number alterations were annotated using the biomaRt package version 2.46.0 (Bioconductor, https://bioconductor.org/, accessed on 20 August 2023) and were queried for “cancer related genes” using the “Cancer Gene Census” in the Catalog Of Somatic Mutations In Cancer (COSMIC) database (GRCh 37 v91) [28].

### 3.2. Characteristics of NCC PMP1-C1 Cells

SNP array analysis revealed amplifications and multiple deletions in the original tumor tissue, and NCC PMP1-C1 cells were detected, which were similar (Figure 2). As copy number variants (CNVs), partial amplifications of chromosomes 1, 2, 7, 8, 9, 11, 12, 15, 16, and 17 and deletions of chromosomes 8, 10, 13, 14, 15, 17, and 20 (Supplementary Table S2) were involved. In NCC-PMP1-C1 cells, among the genes with multiple amplifications and deletions, CNVs of cancer-related genes were not identified. The KRAS c. G35T mutation, which is typical of PMP, was detected in NCC-PMP1-C1 cells (Figure 3). NCC PMP1-C1 cells exhibited a semi-adherent character and pleomorphic cell appearance (Figure 4A,B). The resuspended cells were embedded in paraffin for morphological observation. The consecutive sections were analyzed using H&E (Figure 4C,D) and Alcian blue staining (Figure 4E). The cells exhibited pleomorphic cells with nuclear atypia. The appearances matched the pathological features of the original tumor (Figure 1C,D). Alcian blue staining highlighted thick mucinous material with epithelial cells. The population doubling time of NCC-PMP1-C1 cells was approximately 147 h based on the growth curve (Figure 4F).

## 4. Discussion

Paragraph 2

NCC-PMP1-C1 cells were established from a patient with high-grade PMP that metastasized to the right thigh. The strengths of this study are numerous. First, by collecting epithelial cells and optimizing the culture medium and matrix for PMP, we optimized protocols for the establishment of patient-derived PMP cell lines. Second, without artificial immortalization, such as transfection of the SV40 virus, we established NCC-PMP1-C1 with viability over 25 passages. Previously, two PMP cell lines, N14A and N15A, have been reported as the immortalized cell lines using a lentivirus with the entire SV40 genome, enabling the cells to achieve passaging for over 20 passages [29]. Third, NCC-PMP1-C1 cells comprised the first PMP cell line derived from a metastatic tumor of a patient with PMP. It is well known that PMP usually does not produce metastasis. NCC-PMP1-C1 originated from a distant metastasis and should be considered to be quite aggressive and not representative of the usual biological behavior of PMP. N14A and N15A are derived from primary appendiceal disease tissues [29]. Fourth, to validate NCC-PMP1-C1 cells for tumorigenesis, we injected NCC-PMP1-C1 cells into nude mice intraperitoneally. Cell-line-derived PMP mouse models were established to mimic tumor macro pathological findings, with abdominal distension, tumor nodule formation, mucin production, micro-pathological findings with cellular atypia, and similarity of immunohistochemistry. The cell-line-derived PMP mouse model can be used for the identification of anti-cancer drugs for HIPEC and provide suggestions for novel therapeutic strategies. Finally, the genetic profiles of PMP using SNP array analysis revealed multiple CNVs. Previously, Sio et al. reported MCL1 and JUN amplifications in several PMP samples using panel sequencing [30]. There are no reports of comprehensive copy number analysis with SNP array, CGH, whole exome sequence, or whole genome sequence in PMP.

## Error in Figure

In the original publication [1], there was a mistake in Figure 2B as published. SNP array data on copy number variations was conducted using an inappropriate cell line. Figure 2B of the SNP array data in the original manuscript was replaced with SNP array data conducted with NCC-PMP1-C1 cells after confirmation of their STR data. The corrected Figure 2B and Figure legend of Figure 2B appear below.

**Figure 2:**
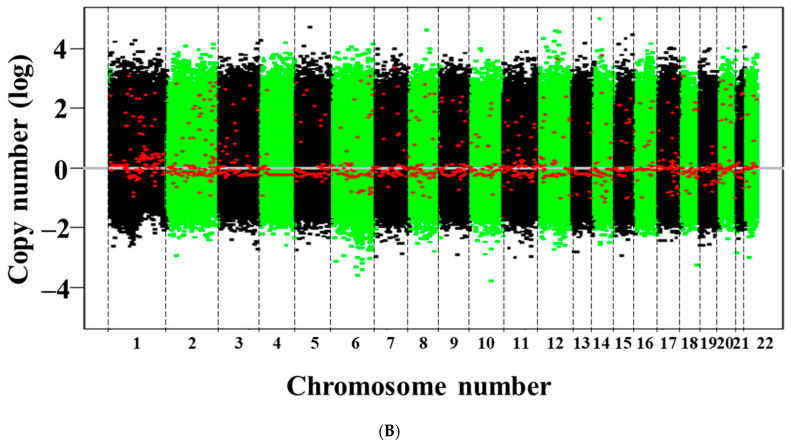
Single nucleotide polymorphism (SNP) array of the original tumor tissue and NCC-PMP1-C1 cells. The profile of copy number variants for the original tumor tissue (**A**) and NCC-PMP1-C1 cells (**B**). The x and y axes indicate the chromosome location and the log ratio of the copy number, respectively. The x and y axes indicate the genomic positions and the copy number, respectively.

The authors state that the scientific conclusions are unaffected. These corrections were approved by the Editor in Chief. The original publication has also been updated.

## References

[1] Noguchi R., Yoshimatsu Y., Sin Y., Ono T., Tsuchiya R., Yoshida H., Kiyono T., Yonemura Y., Kondo T. (2022). Establishment and Characterization of NCC-PMP1-C1: A Novel Patient-Derived Cell Line of Metastatic Pseudomyxoma Peritonei. J. Pers. Med..

