# Supplementary material for: Establishment and Characterization of NCC-PMP1-C1: A Novel Patient-Derived Cell Line of Metastatic Pseudomyxoma Peritonei
Source: J Pers Med. 2022 Feb 10;12(2):258. doi: 10.3390/jpm12020258 (PMC8877412; doi:10.3390/jpm12020258)
Supplement: Supplementary file 1 [file jpm-12-00258-s001.zip › SuppleFigS1_STR_NCC-PMP1-C1.pptx]

## Slide 1
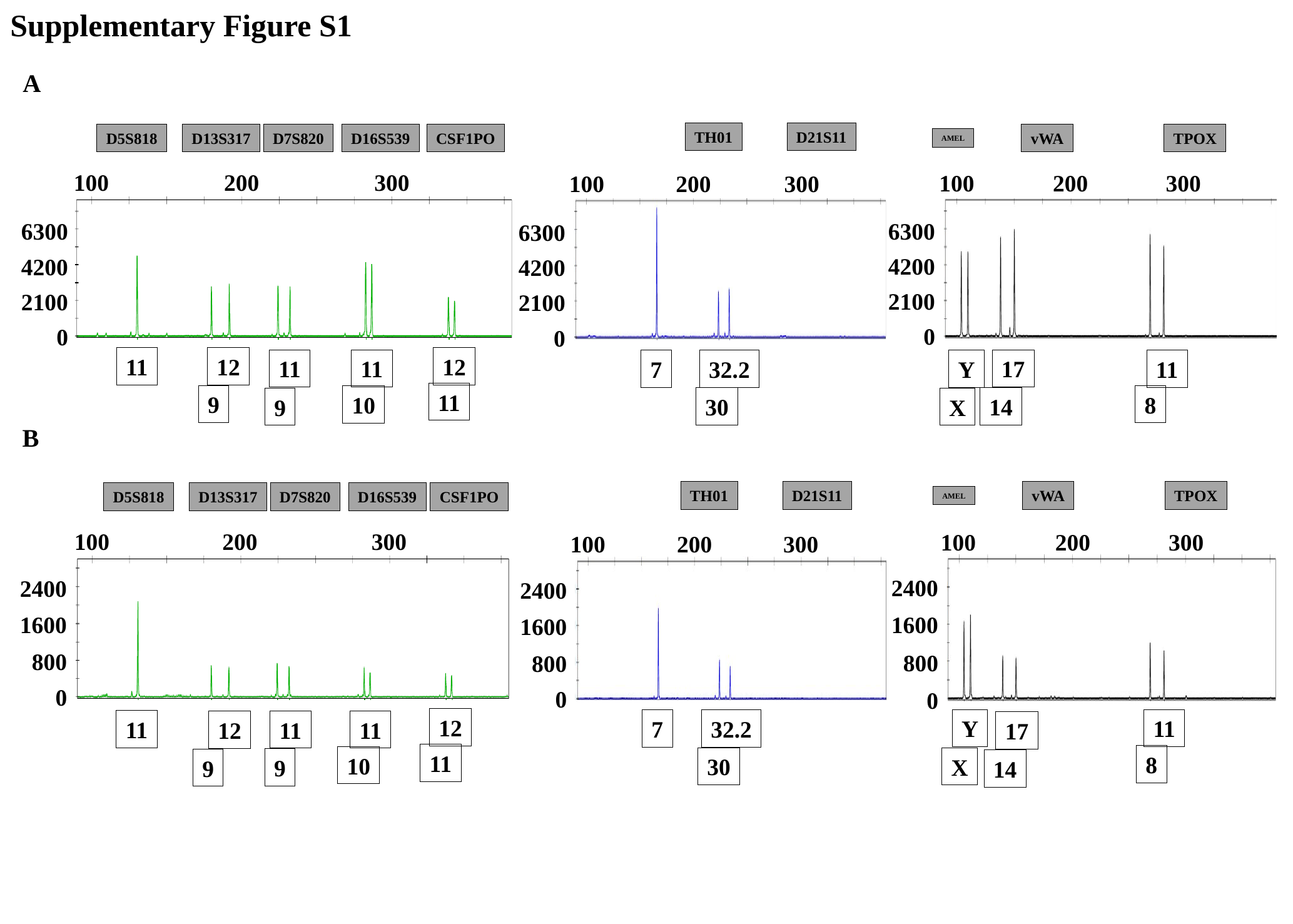

Supplementary Figure S1
A
B
TH01
D21S11
AMEL
vWA
TPOX
D5S818
D13S317
D7S820
D16S539
CSF1PO
100
200
300
100
200
300
100
200
300
6300
4200
2100
0
6300
4200
2100
0
6300
4200
2100
0
12
9
11
11
9
11
10
12
11
17
14
Y
X
11
8
7
32.2
30
TH01
D21S11
AMEL
vWA
TPOX
D5S818
D13S317
D7S820
D16S539
CSF1PO
100
200
300
100
200
300
100
200
300
2400
1600
800
0
2400
1600
800
0
2400
1600
800
0
Y
X
11
8
7
32.2
30
11
11
9
11
10
12
11
12
9
17
14
