# Supplementary material for: Establishment and Characterization of NCC-PMP1-C1: A Novel Patient-Derived Cell Line of Metastatic Pseudomyxoma Peritonei
Source: J Pers Med. 2022 Feb 10;12(2):258. doi: 10.3390/jpm12020258 (PMC8877412; doi:10.3390/jpm12020258)
Supplement: Supplementary file 1 [file jpm-12-00258-s001.zip › SupplementFigS2_HTS_NCC-PMP1-C1.pptx]

## Slide 1
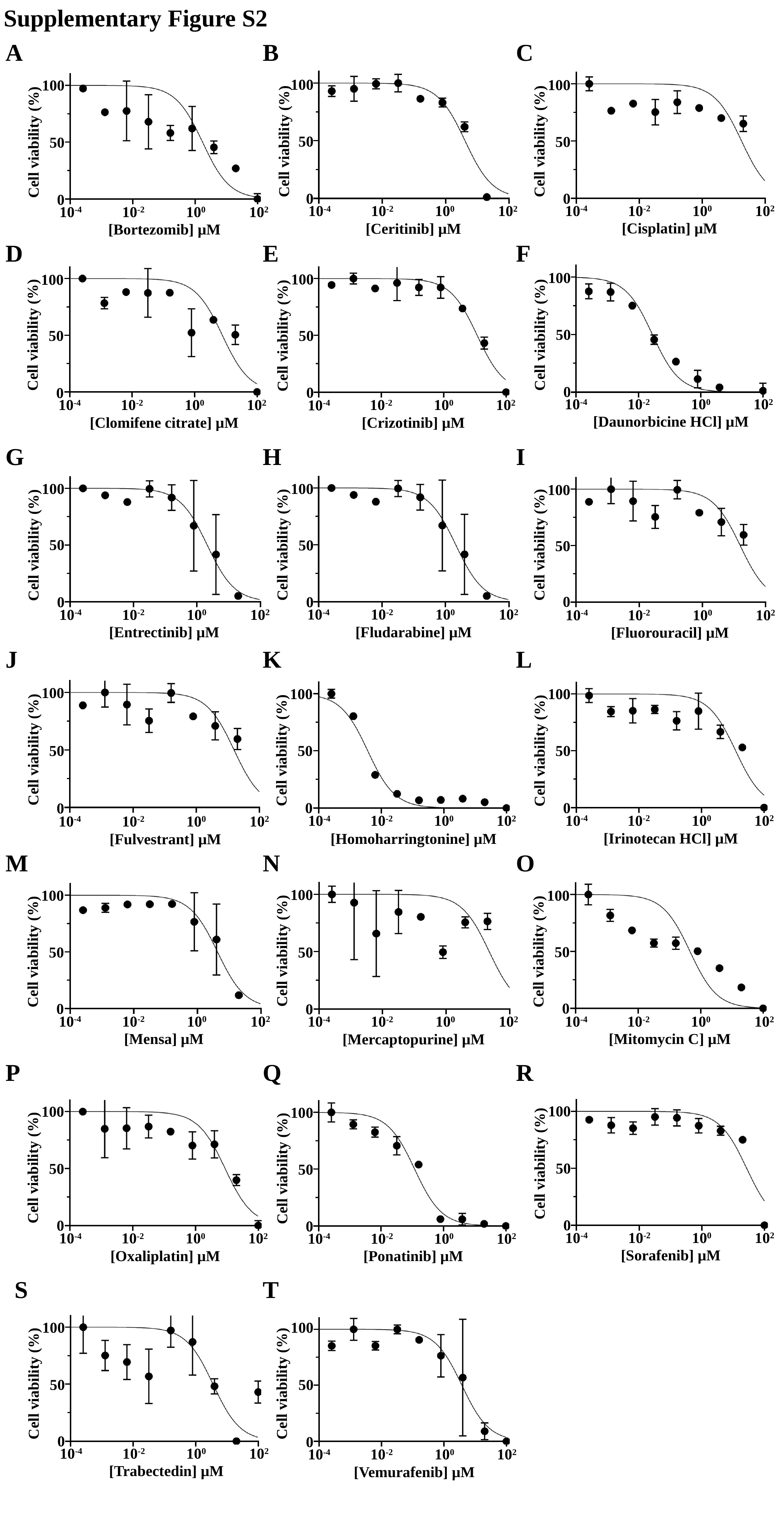

Supplementary Figure S2
A
B
C
100
50
0
100
50
0
100
50
0
Cell viability (%)
Cell viability (%)
Cell viability (%)
10-4
10-2
100
102
10-4
10-2
100
102
10-4
10-2
100
102
[Cisplatin] µM
[Ceritinib] µM
[Bortezomib] µM
D
E
F
100
50
0
100
50
0
100
50
0
Cell viability (%)
Cell viability (%)
Cell viability (%)
10-4
10-2
100
102
10-2
100
102
10-4
10-4
10-2
100
102
[Daunorbicine HCl] µM
[Clomifene citrate] µM
[Crizotinib] µM
G
H
I
100
50
0
100
50
0
100
50
0
Cell viability (%)
Cell viability (%)
Cell viability (%)
10-4
10-2
100
102
10-4
10-2
100
102
10-4
10-2
100
102
[Fludarabine] µM
[Entrectinib] µM
[Fluorouracil] µM
J
K
L
100
50
0
100
50
0
100
50
0
Cell viability (%)
Cell viability (%)
Cell viability (%)
10-4
10-2
100
102
10-4
10-2
100
102
10-4
10-2
100
102
[Irinotecan HCl] µM
[Homoharringtonine] µM
[Fulvestrant] µM
M
N
O
100
50
0
100
50
0
100
50
0
Cell viability (%)
Cell viability (%)
Cell viability (%)
10-4
10-2
100
102
10-4
10-2
100
102
10-4
10-2
100
102
[Mensa] µM
[Mitomycin C] µM
[Mercaptopurine] µM
P
Q
R
100
50
0
100
50
0
100
50
0
Cell viability (%)
Cell viability (%)
Cell viability (%)
10-4
10-2
100
102
10-4
10-2
100
102
10-4
10-2
100
102
[Sorafenib] µM
[Oxaliplatin] µM
[Ponatinib] µM
S
T
100
50
0
100
50
0
Cell viability (%)
Cell viability (%)
10-4
10-2
100
102
10-4
10-2
100
102
[Trabectedin] µM
[Vemurafenib] µM
